# Wound infection and pain one month after trauma: an underestimated threat

**DOI:** 10.3389/fpain.2025.1647785

**Published:** 2025-08-21

**Authors:** Kateryna Ksenchyna, Oleh Ksenchyn, Dmytro Dmytriiev, Oleksandr Nazarchuk

**Affiliations:** National Pirogov Memorial Medical University, Vinnytsya, Ukraine

**Keywords:** chronic pain, superinfection, traumatic wounds, antibiotics, neurotoxicity, limb injuries, combat trauma

## Abstract

**Background:**

Pain is a common complication after combat injuries to the extremities. The role of nerve damage in the development of post-traumatic pain is recognized and described in the literature, superinfection as a potential factor has not been studied sufficiently.

**Objective:**

To establish the relationship between the characteristics of the wound microbiota, the intake of different groups of antibiotics and the development of chronic pain in patients with traumatic injuries of the extremities.

**Methods:**

We conducted a prospective study that included 56 patients. All participants were male, aged 25 years and older. In addition, a mandatory inclusion criterion in the study was the presence of prolonged wound healing, longer than 1 month. We performed a microbiological study of wound contents and assessed the frequency of use of different antibiotics to combat infection. At the same time, pain intensity was assessed using a numerical pain rating scale. Patients were divided into two groups: uncomplicated infection and superinfection. Statistical analysis was performed using *t*-tests, Fisher's exact test, and multiple linear regression.

**Results:**

Superinfection was found in 50% of patients and was significantly associated with higher pain intensity (*p* < 0.01). Based on the results of the regression analysis, superinfection was found to be an independent predictor of pain severity (*β* = 1.31; *p* = 0.001). The use of aminoglycosides and carbapenems showed a trend towards increased pain scores, although statistical significance was not achieved.

**Conclusions:**

Wound superinfection is a distinct predictor of the development of chronic pain after traumatic injury. Early microbiological monitoring and cautious use of neurotoxic antibiotics may reduce long-term pain in affected patients. For a deeper understanding of the processes and factors that contribute to and potentiate the development of pain syndrome, further studies are needed on microbial-neuroimmune interactions, taking into account the duration of antibiotic use and their combinations.

## Introduction

Limb trauma is the most common combat injury with a high incidence of deep soft tissue infection (2, 3).

It is a well-known fact that injuries are one of the significant causes affecting the overall morbidity and mortality rates worldwide. According to the literature, in 2015, the Disability Adjusted Life Year (DALY) rate due to injuries was 11% of the global burden of disease (1).

Non-healing wounds were also a frequent cause of hospitalization among the civilian population in peacetime, but among such patients there were mainly individuals with chronic diseases that reduce the body's resistance and slow down the healing, regeneration processes, etc. The long time healing wounds in young and middle-aged people significantly affect their working capacity, social rehabilitation and the economic state of the state as a whole. Therefore, such wounds were described as a “silent epidemic”. Among the European population as a whole, the diagnostic rate of wounds (a significant proportion of which are colonized by various microbes) was reported to be from 0.3%–0.4% (4–6).

A chronic wound is defined as an acute wound that has not healed within four weeks and does not go through the normal stages of healing. However, some experts prefer to wait 3 months before diagnosing a chronic wound. Factors that worsen the prognosis of patients with wounds include age, immune status, malnutrition, infection, inadequate oxygenation or perfusion, smoking, disease, medications, radiation, and chemotherapy (7, 8). The wound microbiota, and more importantly the type and microbial load of microorganisms, significantly affects the virulence and inflammatory process and the host immune response. Understanding the intricacies of the course of wound infections, mechanisms of microbial defense and aggression, which helps to better understanding the processes occurring and contributes to a more rational use of antibiotics and antimicrobial agents (9).

Many studies have shown that toxins and substances associated with pathogens can affect sensory structures, causing pain. Pain also occurs directly from tissue damage caused by bacterial, fungal, and viral infections. It has been shown that toxins and substances associated with pathogens can affect sensory structures, resulting in pain (10, 11).

Superinfection is a significant issue in modern medicine, particularly concerning rising antibiotic resistance. It complicates the course of the underlying disease, prolongs hospitalization, increases treatment costs, and heightens the risk of fatal outcomes.

It is impossible not to mention the fact that the unreasonable use of antibacterial agents for the prevention of infections, both for minimally invasive and open surgical operations, is an independent factor that increases microbial resistance (12, 13).

Superinfections are particularly prevalent in patients with compromised immunity, such as those recovering from severe injuries, burns, infectious diseases, or those undergoing prolonged treatment with broad-spectrum antibiotics. Early diagnosis and effective treatment necessitate microbiological monitoring and appropriate antimicrobial therapy. Superinfection in this context is defined as the development of a new bacterial infection against the background of an already existing one, with the participation of additional pathogens, which complicates the course of the process and may be associated with an increased risk of antimicrobial resistance, worsening clinical prognosis, chronic pain development.

Chronic pain following trauma is a major source of long-term disability, particularly in combat-injured populations. While structural and neuropathic injury mechanisms are well documented, less is known about the contribution of post-traumatic wound infections. It is important to understand the potential mechanisms of injury, the location of pain, and its severity. In addition, it is important to identify factors that initiate pain (14).

Superinfections can induce inflammatory, ischemic, and pharmacologic insults to peripheral nerves and central pain pathways.

This study **aims** to evaluate how wound microbiota and subsequent antibiotic therapy may influence the development of one month persistent wound pain.

## Material and methods

**Figure 1:**
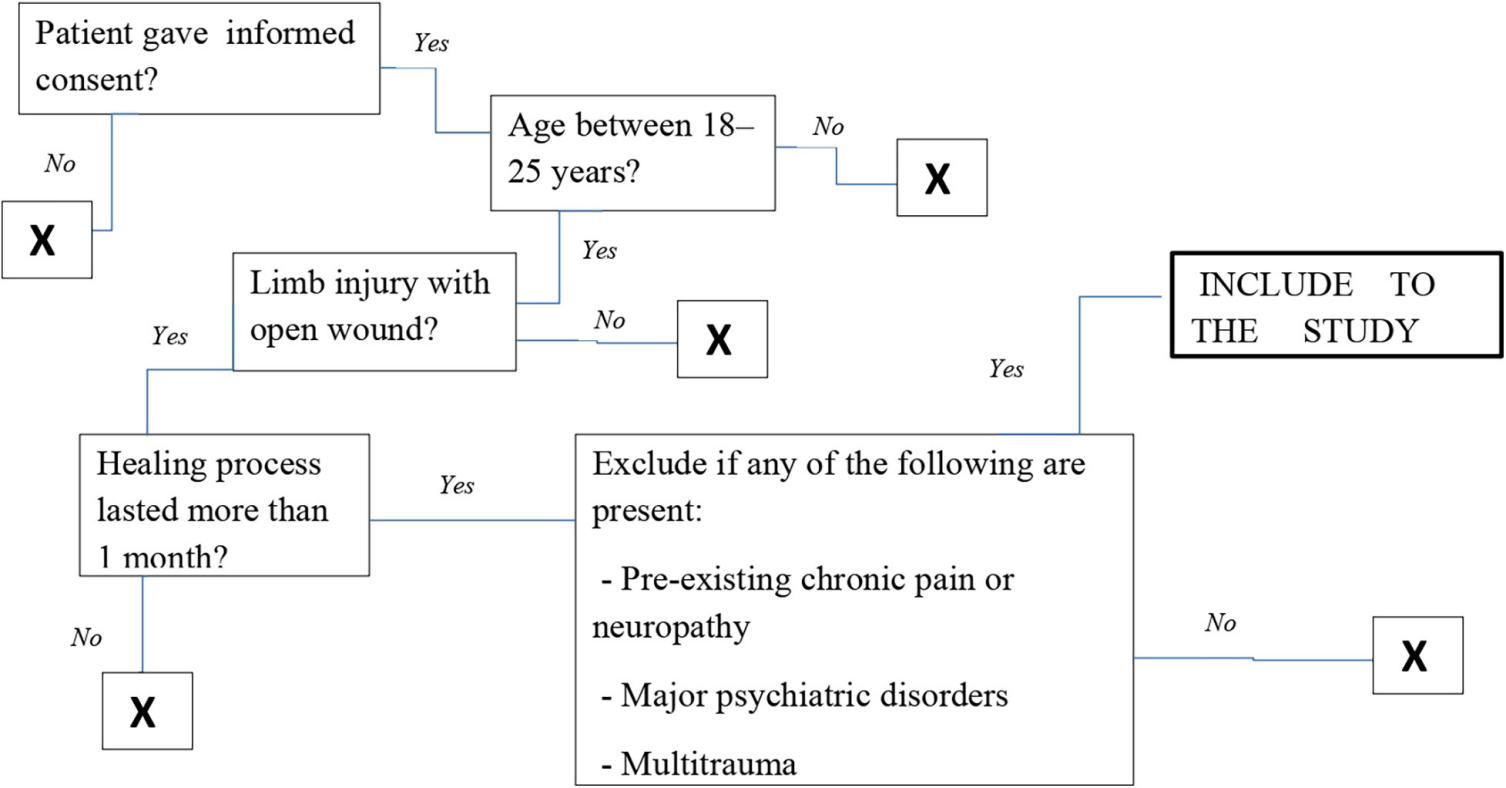
Inclusion criteria to the study.

We conducted a prospective study involving 56 patients who were treated in Municipal Non-profit Enterprise «Vinnytsya Regional Clinical Hospital Vinnytsya Regional Council» This prospective observational cohort study was conducted between 2023 and 2024 in Ukraine. The study followed STROBE guidelines (Figure 1).

### Microbiologocal study

Bacterial cultures were obtained from combat wound soft tissue samples. All patients were informed about the publication of general results without disclosure of personal data. Identification of isolates from bacterial growth colonies was performed according to standard microbiological methods, considering the main cultural characteristics. We gathered soft tissue samples from patients whose wound healing process took between 3 and 6 weeks post-injury. These wounds showed signs of inflammation and either exhibited poor or no signs of epithelialization. We took in account results of microbiological study if bacterial count was higher 10^5^ CFU. Before collecting the material, the wound was washed with sterile saline to remove surface contamination. Direct collection was carried out with a swab of viable tissue (area of 1 cm²), followed by placing the sample in a special container for transportation to the University Microbiology Laboratory of the Pirogov National Medical University, Vinnytsya, along with a completed appropriate form (15).

**Figure 2:**
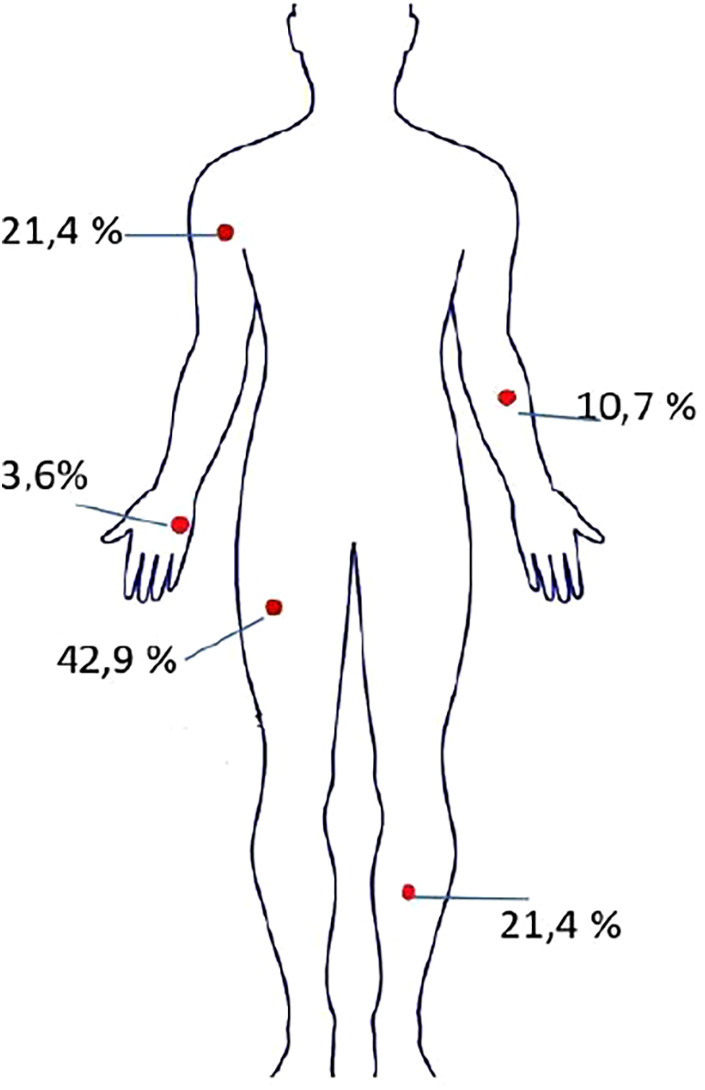
Anatomical distribution of locations for soft tissue sample collection.

In total, soft tissue samples were collected from wound surfaces for microbiological examination from the following locations: upper limbs 35.7% (*n* = 20 з 56) lower limbs 64.3% (*n* = 36 з 56) (Figure 2).

### Group distribution

Based on the results of the microbiological examination, two groups of patients were formed. The first group included individuals with uncomplicated infection. In these patients, a re-examination of the microbial composition of soft tissue wound samples revealed one new type of bacteria, which was different from the pathogen identified in the first days after hospitalization.

The second group included patients in whom a combination of a previously isolated pathogen with new species of bacteria was detected during a re-examination of the microbiological examination. This type of mixed infection was considered a superinfection, where one pathogen potentially contributed to the colonization of the wound by additional microorganisms.

### Pain assessment

Pain was assessed using the Numeric Rating Scale (NRS) scale 1 month after trauma. The numerical pain scale is a subjective method of assessing pain intensity that is widely used in clinical practice (16). The patient is given a visual line from 0–10 to select the number that most accurately reflects the pain sensation at the time of the examination: 0—no pain; 1–3—mild pain (mild, does not interfere with daily activities); 4–6—moderate pain (may affect activity and concentration); 7–9—severe pain (significantly interferes with normal life activities); 10—unbearable pain (maximum possible pain, difficult to tolerate).

### Statistical analysis

Qualitative variables were expressed as absolute numbers and percentages. Quantitative variables were expressed as means and standard deviations or medians and interquartile ranges. Before performing statistical calculations, the normality of the data distribution was checked using the D'Agostino-Pearson test. For normally distributed data, parametric statistical methods such as arithmetic mean, standard deviation, Pearson correlation coefficient and *t*-test were used. In cases of non-normal distribution, we calculated the median (Me) and interquartile ranges, Kendall correlation coefficient. If the total number of cells was less than five, Fisher's exact test was used. We also applied regression analysis (multiple linear regression method) to identify the relationship between pain intensity and the presence of mono-, superinfection and the use of different classes of antibiotics. To evaluate statistical power of an existing study we used *post-hoc* Power Calculator.

The local Committee on Bioethics of National Pirogov Memorial Medical University, Vinnytsya, Ukraine approved all steps of the research and the publication of the results was satisfied (Protocol No. 2; 10.02.2025).

## Results

Baseline data included age, gender, type and location of trauma, and duration until wound closure (Table 1).

**Table 1:** Demographic characteristics of participants.

Variable	Value
Number of patients	56
Age (mean ± SD)	34.16 ± 9.4
Gender (male)	56 (100%)
Character of trauma (*n*)	
Blast injury	18
Burns	24
Amputations	17

We did analysis of the number and percentage of patients depending on the subjective assessment of pain on the NRS. The largest proportion of patients assessed the pain as mild to moderate. Severe and unbearable pain was not recorded (Table 2).

**Table 2:** Distribution of patients by pain intensity level on a numerical scale.

NRS of pain (points)	“0”	“1–3”	“4–6”	“7–9”	“10”
Patients (*n*, %)	2 (3.5%)	24 (43%)	30 (53.5%)	0	0

The next step of the study was to assess the frequency of isolation of isolates of the same type of bacteria or a combination of isolates of several bacteria. We obtained approximately the same frequency of occurrence of uncomplicated infection and superinfection among patientіs, 28 patients (50%) and 28 (50%), respectively. When calculating the D'Agostino-Pearson test, the distribution in both groups was normal: in the group of patients with monoinfection *P* = 0.9183.95% CI for the mean 2.2054–3.2946, in group of patients with superinfection *P* = 0.2953 CI for the mean 3.4985–4.4301.

We used the *post-hoc* Power Calculator to calculate the study's statistical power, which was 93.5% with an alpha (probability of a Type I error) 0.05.

**Figure 3:**
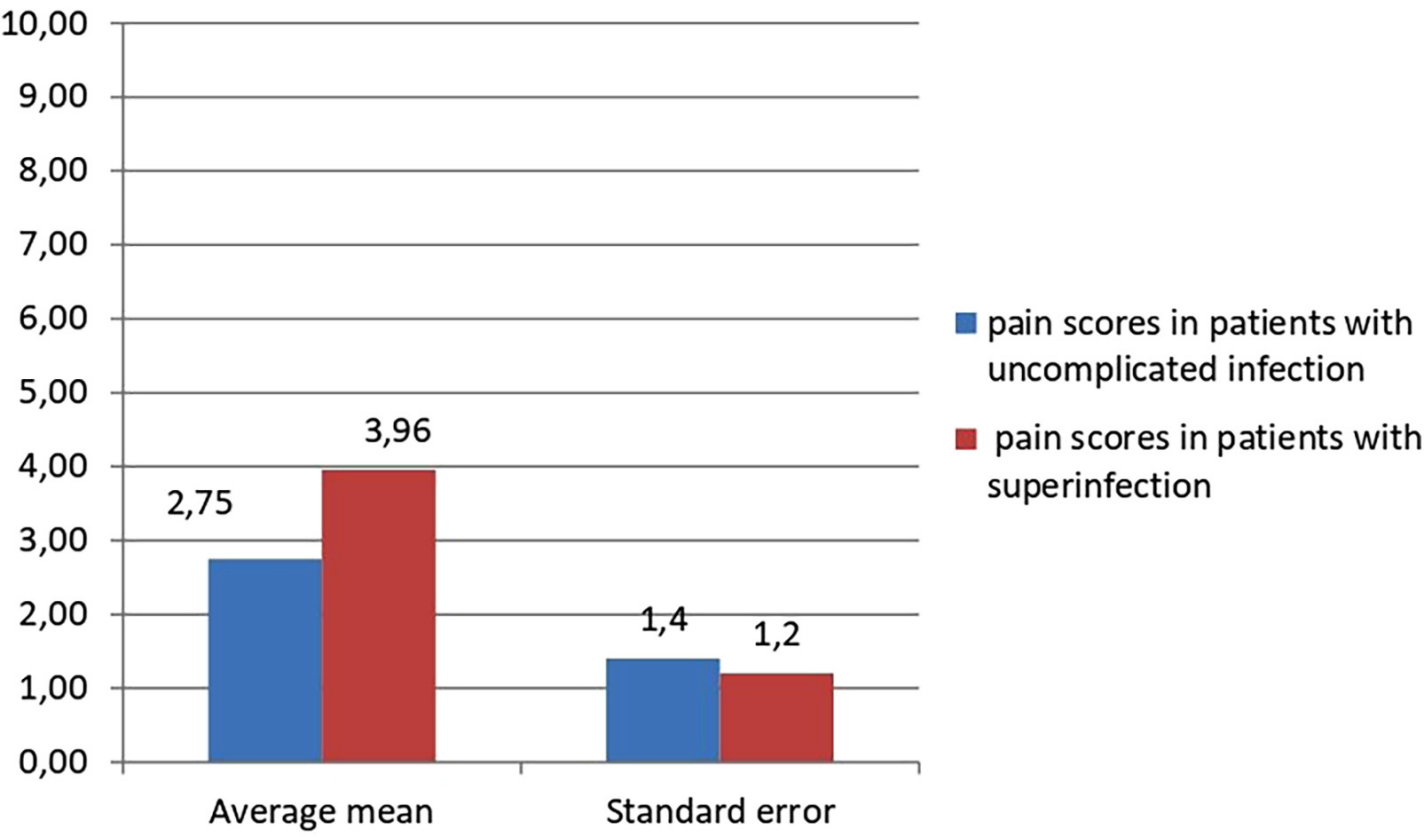
Comparative assessment of pain intensity m patients with uncomplicated infection and in patients with superinfection.

In both groups of patients, we conducted a survey on the severity of pain and its intensity using the NRS scale, calculated the average value of the results obtained. In addition, when further comparing the obtained indicators with each other, we obtained a statistically significant difference between the average values of pain intensity in the group of patients with uncomplicated infection and the average value of pain intensity in patients with superinfection, *p* < 0.01 (Figure 3).

**Figure 4:**
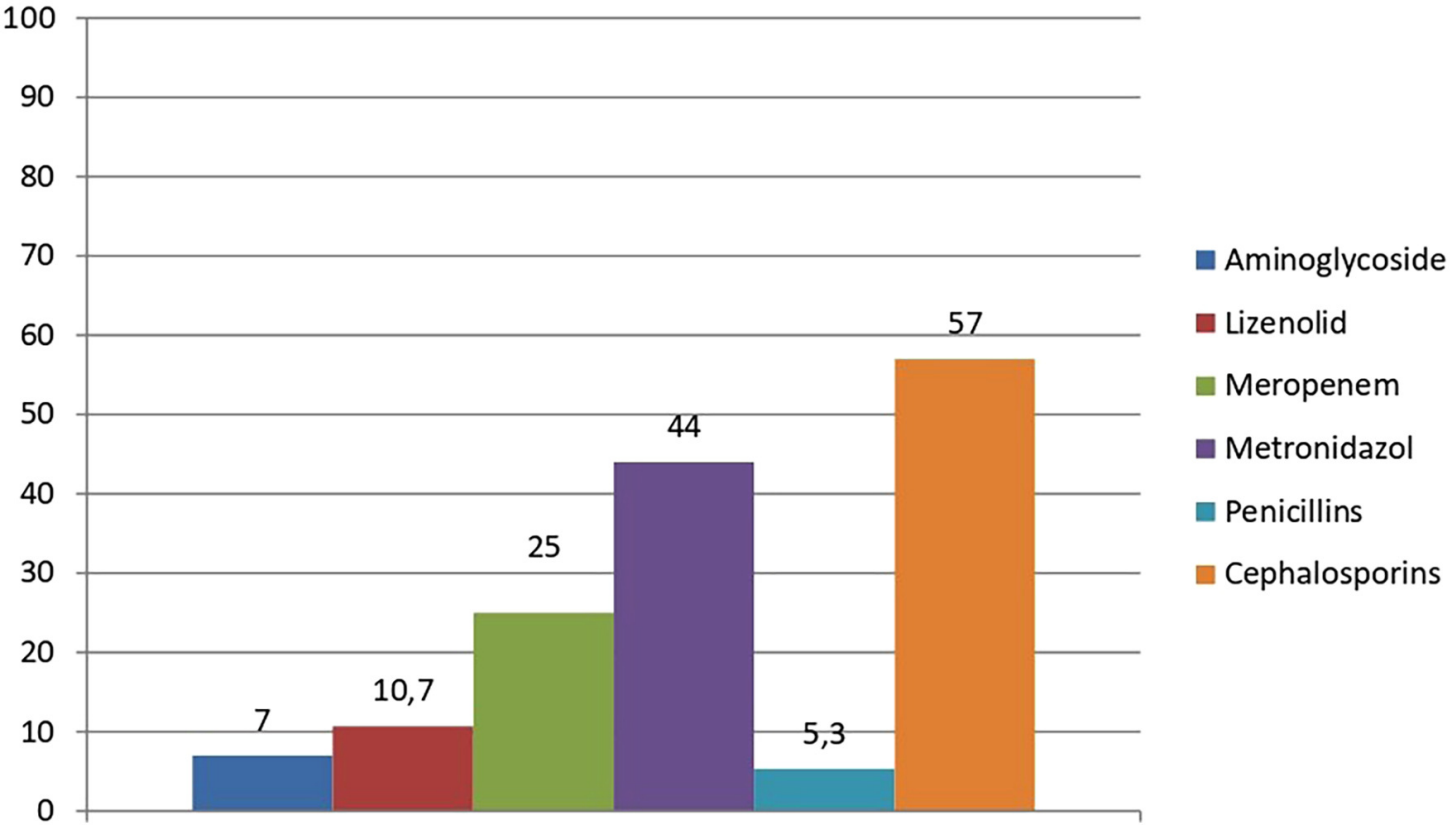
Percentage use of antibiotic groups in patients with infected wounds (% from 56 clinical cases).

We also assessed the frequency of use of different antibiotics in patients for the treatment of wound infections. The bar graph illustrates the frequency of prescription of different groups of antibiotics in percentage among patients. The most frequently used group is cephalosporins, followed by metronidazole and meropenem, and penicillins were prescribed in the least, only in isolated cases (Figure 4).

Furthermore, we applied multiple linear regression analysis to comprehensively evaluate the influence of wound superinfection and the administration of various antibiotic regimens on the severity of pain experienced by patients. This approach allowed us to simultaneously account for multiple independent variables and to determine the extent to which each factor contributed to the overall pain intensity. The presence of superinfection was considered as a categorical variable (yes/no), while antibiotic treatment was categorized based on the class and spectrum of antimicrobial agents used.

As a result, we obtained a statistically significant effect of the presence of wound superinfection on pain intensity: patients with superinfection had higher values according to the NRS. The R² value is 0.2941, which means that 29.41% of the variance in pain intensity (as measured by the NRS scale) is explained by the independent variables in the model (such as superinfection). Although this indicates a moderate level of explanatory power, it leaves a significant remaining variance (70.59%) that can be explained by other factors not included in the model. The F-ratio of 2.8566 with a *p*-value of 0.014 indicates that the overall model is statistically significant. The beta coefficient for superinfection is 1.3935, which is statistically significant (*p* = 0.0004). Therefore, if a patient has a superinfection, their pain intensity (NRS score) is expected to be approximately 1.39 points higher compared to those who do not have it, assuming that all other variables remain constant (Table 3).

**Table 3:** Impact of wound superinfection and antibiotic therapy on pain severity: results from multiple linear regression.

Independent variables	Coefficient	Standart error	r_partial_	t	P
Superinfection	1.3935	0.3668	0.4808	3.799	0.0004
Aminoglycoside	−1.2517	0.7051	−0.2482	−1.775	0.0822
Lizenolid	0.6949	0.5886	0.1680	1.181	0.2436
Meropenem	0.2162	0.4451	0.06994	0.486	0.6293
Metronidazol	−0.1482	0.3693	−0.05784	−0.401	0.6899
Penicillins	0.3484	0.8450	0.05941	0.412	0.6819
Cephalosporins	0.06976	0.4064	0.02477	0.172	0.8644
Constant—2.6181					
F-ratio = 2.8566; R^2^ = 0.2941; *p* = 0.014					

## Discussion

In this study, we found a statistically significant relationship between the presence of wound superinfection in patients with combat injuries of the extremities and the intensity of pain one month after injury. The results confirmed the hypothesis that the features of the course of infectious complications, in particular superinfection, play an important role in the formation of persistent pain syndrome and its severity, which is probably due to a long-term active inflammatory process, the production of microbial toxins and damage to peripheral nerve structures.

Previously published studies have focused on the neuropathic component of post-traumatic pain, which is directly related to mechanical nerve damage (17).

In our previous studies of possible associations between different characteristics of the wound microbiota and the formation of chronic pain, we found a trend towards higher mean pain scores and a predominance of Gram-negative bacteria in the group of people with amputated limbs compared to the groups of patients with burns and traumatic injuries (*p* > 0.05). Although the correlation analysis between pain intensity and contamination with Gram-negative bacteria did not demonstrate an association between these two variables (18).

However, the results of this study demonstrated that the presence of multiple pathogens in the wound correlated with higher NRS pain scores compared to patients in whom only uncomplicated monoinfection was detected.

The results of the regression analysis also indicate the possible influence of individual classes of antibiotics in the modulation of prolonged pain syndrome, but statistical significance was not achieved. At the same time, the trends regarding the association of aminoglycosides and increased pain intensity are consistent with the literature data on their neurotoxicity.

In their work, the group of researchers *Ajibola* et al. *(2023)* noted a gradual increase in the number of reports of neurotoxicity caused by cefepime, mainly in patients aged 65 years and older (52%). The most common indications for its use were bone and joint infections (25%), urinary tract infections, and pneumonia (22.7% each). Signs of neurotoxicity usually appeared on days 2–5 of treatment, despite the high efficacy of cefepime against gram-negative flora, in particular P. Aeruginosa (19).

At the same time, according to the results of the study by *Mende* et al. *(2022)* during the war in Iraq and Afghanistan, Escherichia coli was the most common gram-negative bacilli and Enterococcus spp. were isolated in 53% of wound samples, and 65% showed growth of other pathogens from the ESKAPE group (20).

Other studies have shown that neurotoxic effects can also be caused by other groups of antibiotics, including aminoglycosides, tetracyclines, macrolides, clindamycin, polymyxins, as well as anti-tuberculosis drugs such as ethambutol, isoniazid, and chloramphenicol (21).

From a clinical perspective, these results indicate the feasibility of early microbiological monitoring, rational choice of antibiotics taking into account potential neurotoxicity, and the implementation of a multidisciplinary approach to the management of patients with a high risk of chronic pain after an infectious complication of trauma.

The limitations of our study are the relatively small sample size, gender homogeneity (all patients were male), and the lack of data on pain intensity over a longer period of time. The study does not account for a number of important factors, such as other medical conditions of the patients (e.g., chronic diseases, mental health, type and duration of pain medication), that can affect both the composition of the microbiome and the perception of pain. Taking these factors into account could improve the accuracy of the results and also help reduce the influence of these variables on the conclusions. The study focuses on the impact of superinfection and antibiotic classes on pain intensity, but does not provide sufficient information on other factors that may influence the outcome. For example, it would be important to assess how the duration and timing of antibiotic treatment affect the change in pain, as these factors can alter the response to therapy.

The study only included patients with wounds that had healed within 3–6 weeks, which may limit understanding of the long-term effects on the microbiome and pain intensity. A study with a longer follow-up period could provide a more complete picture of the changes in microbial composition and their impact on patients' condition in the long term. In further studies, it is advisable to assess the dynamics of the pain syndrome over a period of 3–6 months, as well as to involve biomarkers of neuroinflammation and neuroimaging techniques for a deeper understanding of the mechanisms.

## Conclusion

Superinfection is a major modifiable risk factor for prolonged acute post-traumatic pain. The results of the analysis of the frequency of use of various antibiotics in the treatment of infected wounds or wound complications may indicate a preference for broad-spectrum or reserve antibiotics, which requires further analysis of the feasibility and impact on the development of antibiotic resistance. Preventive strategies should include early infection control, judicious use of neurotoxic antibiotics, and proactive pain monitoring in high-risk patients.

## Data Availability

The original contributions presented in the study are included in the article/Supplementary Material, further inquiries can be directed to the corresponding author.
